# Comparative pharmacokinetics of total ginsenosides between young and aging mice

**DOI:** 10.3389/fphar.2026.1844832

**Published:** 2026-06-30

**Authors:** Zuoyang Li, Nanqi Hou, Yuying Zhou, Yan Zhang, ZiChen Tian, Xinyue Cao, Daqing Zhao, Hang Su, Xiangyan Li

**Affiliations:** 1 Northeast Asia Research Institute of Traditional Chinese Medicine, Jilin Provincial Key Laboratory for Efficacy Research and Utilization of Characteristic Traditional Chinese Medicine, Changchun University of Chinese Medicine, Changchun, China; 2 Department of Endocrinology, The First Affiliated Hospital of Changchun University of Chinese Medicine, Changchun, China

**Keywords:** ginsenosides, MRM, pharmacokinetics, tissue distribution, UPLC-MS/MS

## Abstract

**Introduction:**

Ginsenosides, the primary bioactive constituents of ginseng, are widely recognized as popular natural products with anti-aging properties. However, they generally exhibit poor oral bioavailability. Moreover, existing pharmacokinetic studies have predominantly focused on young animal models, leaving a critical gap in understanding how aging alters the absorption, distribution, metabolism, and excretion of these compounds. This study aims to elucidate the multi-component pharmacokinetics and tissue distribution profiles of total ginsenosides following oral administration in both young and aging mice.

**Methods:**

The validity of the aging mouse model was first confirmed through a series of physiological evaluations. A multiple reaction monitoring (MRM) technique was then employed to conduct comparative pharmacokinetic and tissue distribution analyses of 15 targeted ginsenosides after oral administration in young and aging mice.

**Results:**

Aging mice exhibited significantly higher systemic exposure (AUC) and markedly reduced clearance rates compared to their young counterparts. Tissue distribution analysis revealed the highest accumulation of ginsenosides in the liver, followed by the heart, kidneys, spleen, lungs, testes, and brain, confirming the liver as the primary metabolic site.

**Discussion:**

These findings demonstrate that aging increases systemic exposure, prolongs half-lives, and enhances tissue accumulation of ginsenosides. This study provides essential scientific evidence for the rational clinical application and dosage optimization of ginseng in aging populations.

## 1 Introduction

The process of aging is characterized by a gradual decrease in physical and physiological abilities, leading to an elevated risk of chronic diseases, such as neurodegenerative diseases, metabolic disorders and cardiovascular diseases (Kritsilis et al., 2018; Montégut et al., 2024; Li et al., 2025). This has intensified the search for safe and effective anti-aging interventions, with natural products garnering considerable attention. Among them, ginseng (*Panax ginseng* C.A. Meyer) holds a prominent place in traditional medicine for its ability to promote health and longevity. The primary active constituents of ginseng are total ginsenosides, which particularly demonstrate a variety of pharmacological activities, including anti-inflammatory, antioxidant, and anti-tumor effects (Kim et al., 2012; Lü et al., 2012; So et al., 2013; Qu et al., 2015; Wang et al., 2017). Despite the therapeutic promise of total ginsenoside extracts, its clinical application is limited by the low oral bioavailability of naturally occurring ginsenosides, which is typically less than 10% in rodents (Won et al., 2019). This poor absorption is attributed to high molecular weights, bulky sugar moieties, and extensive biotransformation by gut microflora (Park, 2024). Specifically, major ginsenosides (e.g., Rb1, Rd) are hydrolyzed stepwise by colonic bacteria into rare but more absorbable and active metabolites, such as Compound K (C-K), 20(S)-PPD, and 20(S)-PPT (Yang et al., 2012; Kim, 2013; Sharma and Lee, 2020). While the chemical profiling of ginseng has been extensively studied (Liu et al., 2022; Wang et al., 2024), there remains a critical gap in our understanding of its *in vivo* absorption processes, particularly in the target demographic for aging populations. Most existing pharmacokinetic (PK) studies have focused on single or few components in young animal models, such as Rg3, Rh2, Rb2, leaving a significant gap in our understanding of the multi-component PK profiles in aging populations (Xie et al., 2005; Gu et al., 2009; Miao et al., 2022). This approach fails to reflect the clinical reality where ginseng is consumed as a multi-component extract by aging populations. Elderly individuals experience a 20%–50% decrease in hepatic blood flow. For drugs with high first-pass extraction like propranolol, this reduction in flow directly lowers systemic clearance (Kim et al., 2022). Given that the metabolism and absorption of ginsenosides are highly dependent on gut microflora and hepatic clearance, the PK profiles derived from young models may not accurately represent the drug disposition in the elderly.

Therefore, relying on existing data from young subjects may lead to suboptimal or unsafe dosing strategies for aging mice. To bridge this gap, it is necessary to characterize the overall pharmacokinetic profile of major absorbed ginsenoside components following the administration of total ginsenosides in aging models. This study aims to systematically compare the multi-component PK profiles of total ginsenosides between young and aging mice. By elucidating the age-related differences in the absorption and distribution of these active compounds, this research will provide a crucial scientific basis for the rational clinical application and dosage optimization of ginseng in the aging population.

## 2 Methods

### 2.1 Chemical reagents and materials

22 ginsenoside reference standards (purity ≥98%), including ginsenoside Ro, Re, Rf, Rg1, Rg2, Rg3, Rb1, Rb2, Rb3, Rc, Rd, Ra1, Rk1, Rg5, F5, F1, F2, Pf11, Rh1, 20-O-Glc-Rf, Protopanaxatriol (PPT) and Compound K, were purchased from Chengdu Desite Biotechnology Co., Ltd (Chengdu, China), and internal standard (IS) of digoxin was purchased from Sigma-Aldrich (St. Louis, MO, United States of America). Methanol and acetonitrile were obtained from Fisher Scientific (Pittsburgh, PA, United States of America), and formic acid was purchased from Thermo Scientific (Waltham, MA, United States of America).

### 2.2 Animals

Male C57BL/6 J mice (4-month-old, weighing 26–30 g) were acquired from Changchun YISI Experimental Animal Co., Ltd (Changchun, China, Animal, Quality Certificate No SCXK (JI)-2020–0002). The mice were maintained under a 12 h light-dark cycle at temperature of 25 °C ± 2 °C and relative humidity of 55% ± 5%. The study protocol was approved by the ethical committee of Laboratory Animals at Changchun Chinese Medicine University (Permit Number: 20–008). The animals were divided into the young mice (4-month-old, 6 mice) and aging mice (18-month-old, 6 mice).

### 2.3 Preparation of standard solutions and working solutions

Stock solutions were prepared separately in methanol at a concentration of 5 mg/mL for analytes, while ginsenoside Ra1, Rb1 and Rd were prepared at a concentration of 10 mg/mL. Appropriate volumes of the 22 individual ginsenoside stock solutions were transferred into a 1.5 mL centrifuge tube to prepare a mixed standard solution at a concentration of 100 μg/mL. This mixed standard solution was serially diluted with 50% methanol in water (v/v) to obtain a series of calibration working solutions at concentrations of 1,600, 800, 400, 200, 100, 50, 25, 12.5, 6.25, 3.13, 1.56, and 0.78 ng/mL. Digoxin was added as an internal standard to all working solutions at a final concentration of 100 ng/mL.

### 2.4 Quantitative mass spectrometry analysis

For pharmacokinetic and tissue distribution studies, quantification was conducted using multiple reaction monitoring (MRM) on QTRAP 6500+ (AB SCIEX, Concord, ON, Canada). Components separation was conducted on a CAPCELL PAK ADME column (2.1 mm × 100 mm, 2 μm, OSAKA SODA CO., Japan). The mobile phase consisted of 0.05% formic acid in water (A) and acetonitrile (B), with a gradient elution program as follows: initial 28% B; 0–3 min, 28% B; 9 min, 43% B; 12 min, 71% B; 15 min, 79% B; 16–21 min, 95% B; 21.1–23 min, 28% B. The flow rate was 0.3 mL/min, column temperature was maintained at 35 °C, injection volume was 2 μL, and the autosampler temperature was kept at 4 °C throughout the analysis.

An electrospray ionization (ESI) source was operated in both positive and negative modes with a spray voltage of 5500 V and −4500 V, respectively. The nebulizer and heater gas were both set at 50 psi, and the probe temperature was maintained at 500 °C. Specific mass transitions for 15 ginsenosides and IS are summarized in Table 1. Specific mass transitions for the other 7 ginsenosides are summarized in Supplementary Table S1.

**TABLE 1:** Optimized multiple reaction monitoring (MRM) parameters.

Compound	Module	Precursor ion (*m/z*)	Produc tion (*m/z*)	DP	CE	EP	CXP
Rb1	Negative	1,107.8	945.7	−157	−65	−10	−13
Rb2	Negative	1,123.6	1,077.6	−80	−38	−10	−13
Rb3	Negative	1,123.6	1,077.6	−80	−38	−10	−13
Rc	Negative	1,123.6	1,077.6	−80	−38	−10	−13
Rd	Negative	991.5	621.6	−135	−60	−10	−13
Ra1	Negative	1,209.5	1,077.1	−260	−67	−10	−13
Rg3	Negative	829.5	783.6	−80	−30	−10	−13
CK	Negative	667.2	621.3	−70	−28	−10	−13
Re	Negative	991.5	945.5	−135	−34	−10	−13
Rf	Negative	799.5	475.5	−230	−57	−10	−13
Rg1	Negative	845.5	637.5	−30	−44	−10	−13
Rg2	Negative	829.5	783.6	−80	−30	−10	−13
Rg5	Negative	811.5	765.5	−70	−26	−10	−13
Rk1	Negative	811.5	765.8	−70	−27	−10	−13
Ro	Negative	955.5	793.5	−200	−67	−10	−13
Digoxin	Positive	781.4	651.4	210	10	15	6

Declustering potential (DP), entrance potential (EP), collision energy (CE), and cell exit potential (CXP) for 15 ginsenosides and the internal standard (IS) are listed.

### 2.5 Preparation of sample solutions

Total ginsenosides (TGs) were purchased from Shanghai Yuanye Bio-Technology Co., Ltd (Shanghai, China). The 2 g of TGs were weighed and dissolved in 40 mL of a 0.5% CMC-Na solution for oral administration to mice with a concentration of 50 mg/mL and given to mice by oral gavage at a dose of 200 mg/kg.

### 2.6 Processing of plasma samples and tissue samples

A total of 200 μL of blood samples were collected from the ophthalmic veins in heparinized microcentrifuge (EP) tubes at 0, 0.083, 0.25, 0.5, 1, 3, 6, 12, 24, 36, 48, and 72 h after dosing the TGs (n = 6 per group; young and aging mice). Aliquots (100 μL) of plasma were transferred into a 1.5 mL EP tube and spiked with 5 μL of the IS solution (2 μg/mL digoxin). An aliquot of 100 μL of the plasma sample was deproteinized by adding 400 μL of methanol/acetonitrile (1:1, v/v) containing 5 μL of IS (2 μg/mL). For tissues, 10 mg samples were homogenized in 100 μL of physiological saline; then, an aliquot (100 μL) of the tissue homogenate was added 350 μL methanol/acetonitrile (1/1, v/v) and 50 μL of IS to the tube. All mixtures were vortexed for 1 min and centrifuged at 14,000 g for 15 min at 4 °C. The resulting supernatants were evaporated to dryness at 25 °C, reconstituted in 100 μL of 50% methanol, and centrifuged again. Finally, 2 μL of the supernatant was injected for UPLC-MS/MS analysis.

### 2.7 Evaluation of the natural aging mice model

To validate the natural aging mice model, grip strength tests, novel object recognition (NOR) tests, body composition analysis, and echocardiography were employed to evaluate the aging mice model. Besides, the superoxide dismutase (SOD) activity, malondialdehyde (MDA) levels and β-galactosidase (Gal) activity were detected in heart, liver and brain to evaluate the aging model. These evaluations ensured the suitability of the 18-month-old mice as a natural aging model.

### 2.8 Method validation

Method validation was conducted in accordance with the FDA Guidance for Industry on Bioanalytical Method Validation, assessing the following parameters: linearity, limit of quantification (LOQ), precision, accuracy, matrix effect, and stability.

### 2.9 Data analysis

The peak areas of the target analytes were imported into the standard curve, and the concentrations were calculated using SCIEX OS software. Pharmacokinetic parameters were subsequently calculated based on a non-compartmental model using Phoenix WinNonlin 8.1 software. The concentration data were imported into GraphPad Prism 8.3 to plot plasma concentration-time profiles and histograms, and were presented as mean ± standard deviation (SD). Comparisons of pharmacokinetic parameters between young and aging mice (n = 6 per group) were performed using one-way analysis of variance, with pairwise comparisons between groups conducted using the t-test. *P* < 0.05 was considered statistically significant.

## 3 Results

### 3.1 Phenotypic validation of the natural aging mouse model

**FIGURE 1:**
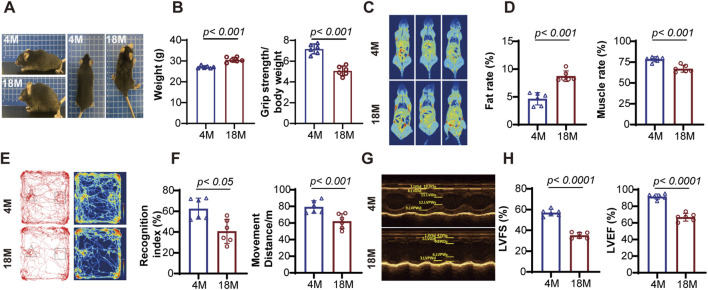
Phenotypic characterization in young versus aging mice **(A)** Representative photographs of young and aging mice **(B)** Body weight and grip strength **(C)** Representative axial magnetic resonance (MR) images **(D)** Body composition analysis **(E)** Trajectory heatmaps from the novel object recognition (NOR) test **(F)** NOR index and total motion distance **(G)** Representative echocardiograms assessing cardiac systolic function. Representative axial magnetic resonance (MR) images **(H)** Quantification of left ventricular ejection fraction (LVEF) and fractional shortening (LVFS).

Before comparing the pharmacokinetic profiles of ginsenosides, we first validated the aging phenotype of 18-month-old aging mice relative to 4-month-old young mice. Morphologically, during the feeding period, young mice were observed to be more active and agile compared with 18-month-old mice. Young mice exhibited healthier skin and coat condition, whereas aging mice showed dull fur, hair loss, and skin laxity (Figure 1A). This was accompanied by a significant increase in body weight compared to the young mice (Figure 1B, *P* < 0.001). Body composition and imaging analyses further revealed that this weight gain in aging mice was driven by significantly increased fat deposition in the abdominal and perigonadal regions, alongside a marked reduction in lean mass (Figures 1C,D, *P* < 0.001). Behaviorally and physically, aging mice displayed a diminished capacity. They exhibited a significant reduction in relative grip strength and total distance traveled, reflecting an age-related decline in muscle function and physical activity (Figures 1B,F *P* < 0.001). Furthermore, in the novel object recognition (NOR) test, aging mice showed a significantly lower preference for exploring the novel object, indicating impaired recognition memory (Figures 1E,F, *P* < 0.001). At the organ and cellular levels, Cardiac function assessment revealed significantly impaired systolic function in aging mice, as evidenced by marked reductions in left ventricular ejection fraction (LVEF) and left ventricular fractional shortening (LVFS) (Figures 1G,H, *P* < 0.0001). Biochemically, aging mice suffered from severe oxidative stress and cellular senescence in major organs (heart, liver, and brain). This was characterized by significantly decreased superoxide dismutase (SOD) activity, elevated malondialdehyde (MDA) levels, and a significant upregulation of senescence-associated β-galactosidase (β-Gal) activity compared to young mice (*P* < 0.01, Supplementary Figure S1). In summary, these multifaceted morphological, behavioral, and biochemical alterations successfully validate the 18-month-old C57BL/6 J mice as a robust natural aging model for our subsequent pharmacokinetic studies.

### 3.2 Methods validation

**FIGURE 2:**
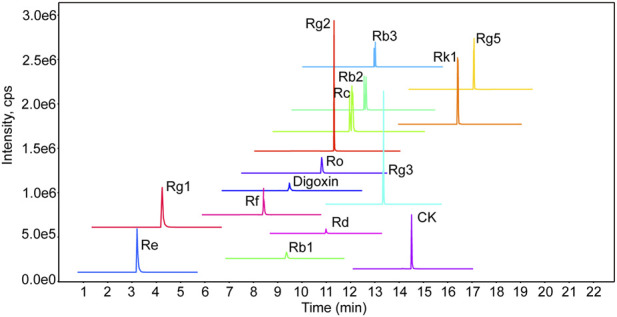
The extracted ion chromatograms of the 15 ginsenosides and IS.

The results for linearity and the limit of quantification (LOQ) in plasma are presented in Table 2. The results for linearity and the limit of quantification (LOQ) in blank solution are presented in Supplementary Table S2. In addition, the MRM channels for the 15 ginsenosides are shown in Figure 2. The calibration curves showed excellent linearity (*R* ^2^ > 0.99) for all ginsenosides. Accuracy, intraday/interday precision, recovery, and matrix effect data are summarized in Table 3. Both intraday and interday precision met the acceptance criterion (RSD <15%). Accuracy ranged from 87.6% to 114.9%, within the 85%–115% threshold. Recoveries (85%–115%) and matrix effects (85%–115%) for all ginsenosides met validation criteria, with RSD values < 15%. Stability was evaluated using low/medium/high QC samples under the following conditions: (1) room temperature (4 h), (2) autosampler (4 °C, 7 days), (3) −80 °C freezer (≥30 days), and (4) three freeze-thaw cycles. Sample concentrations were measured after each treatment (Table 4). All results demonstrated satisfactory stability across concentrations, confirming robustness under the tested conditions.

**TABLE 2:** Linearity and Limit of Quantification of 15 ginsenosides.

Analytes	Linearity	*R* ^2^	Linearity	LOQ (ng)
Rb1	y = 0.16392 x + −0.00417	0.99458	3.13–1,600	3.13
Rb2	y = 4.75205 x + 0.00920	0.99462	1.56–800	1.56
Rb3	y = 6.1804 x + −0.01031	0.9923	1.56–800	1.56
Rc	y = 8.57053 x + −0.00868	0.9962	1.56–800	1.56
Rd	y = 0.43384 x + −0.00374	0.9931	12.5–1,600	12.5
Ra1	y = 0.04363 x + 0.00749	0.99695	12.5–800	12.5
Rg3	y = 26.42540 x + 0.09954	0.99708	1.56–800	1.56
CK	y = 6.19056 x + −0.07534	0.99446	12.5–800	12.5
Re	y = 16.90436 x + −0.00466	0.99106	1.56–800	1.56
Rf	y = 0.34560 x + −0.01201	0.99808	12.5–800	12.5
Rg1	y = 6.55300 x + −0.01512	0.99355	1.56–800	1.56
Rg2	y = 40.57173 x + 0.01646	0.99483	1.56–800	1.56
Rg5	y = 32.38406 x + 0.00593	0.99244	1.56–800	1.56
Rk1	y = 19.38168 x + −0.04222	0.99398	1.56–800	1.56
Ro	y = 1.93436 x + −0.00581	0.99486	1.56–800	1.56

**TABLE 3:** Precision, Accuracy, Recovery Rate, and Matrix Effects in the Detection of 15 ginsenosides in plasma.

Analytes	Spiked QC (ng/mL)	Intraday		Interday		Recovery (%)	Matrix effect (%)
		Precision (RSD, %)	Accuracy (%)	Precision (RSD, %)	Accuracy (%)		
Rb1	25	5.32	104.33	5.44	102.76	102.76 ± 5.81	101.68 ± 3.75
	200	3.97	101.79	9.87	92.94	92.94 ± 3.84	98.77 ± 10.45
	1,600	2.42	106.35	5.56	102.37	102.37 ± 7.74	109.56 ± 1.75
Rb2	12.5	6.79	100.16	3.27	99.59	107.43 ± 2.14	109.09 ± 1.85
	100	5.06	99.14	3.13	104.86	82.03 ± 3.16	94.09 ± 4.71
	800	5.76	99.37	2.87	95.56	110.14 ± 5.94	100.79 ± 8.52
Rb3	12.5	5.92	100.55	4.2	103.7	99.16 ± 3.64	101.44 ± 6.44
	100	6.47	100.62	2.08	102.1	66.4 ± 10.24	87.11 ± 6.85
	800	4.73	107.43	2.59	91.36	106.24 ± 3.83	114.68 ± 5.68
Rc	12.5	4.44	90.64	5.23	102.97	110.17 ± 5.73	112.23 ± 2.42
	100	7.08	102.05	2.27	101.27	80.24 ± 7.64	94.36 ± 1.64
	800	6.33	103.55	3.85	92.35	104.31 ± 8.44	100.64 ± 4.81
Rd	25	4.22	100.17	5.3	100.06	112.42 ± 6.42	105.21 ± 8.04
	200	7.72	97.16	3.22	99.29	99.9 ± 8.64	88.57 ± 10.78
	1,600	2.6	103.81	2.56	100.65	99.21 ± 2.45	104.15 ± 5.85
Ra1	25	8.56	100.88	9.71	100.45	110.21 ± 10.4	105.57 ± 1.74
	200	3.27	107.67	3.84	94.68	99.41 ± 3.13	102.47 ± 2.75
	1,600	5.23	113.29	1.49	104.87	105.31 ± 1.34	113.1 ± 4.85
Rg3	12.5	20.3	86.46	4.27	108.39	136.54 ± 7.41	101.88 ± 6.43
	100	3.34	100.77	1.61	103.28	96.43 ± 1.52	93.6 ± 7.59
	800	4.48	100.87	2.55	89.4	114.77 ± 6.41	103.45 ± 2.53
CK	12.5	4.78	102.49	4.96	99.96	109.88 ± 6.13	113.98 ± 7.41
	100	6.26	105.46	3.3	100.52	78.46 ± 4.13	89.42 ± 5.95
	800	10.68	108.65	4.62	99.52	106.06 ± 7.41	102.92 ± 3.65
Re	12.5	10.11	97.6	3.07	100.04	110.83 ± 5.23	108.07 ± 6.42
	100	5.51	103.71	2.99	99.51	70.55 ± 2.14	93.84 ± 2.83
	800	10.07	103.95	2.64	100.45	111.58 ± 8.14	101.98 ± 8.58
Rf	12.5	17.12	110.41	5	100.41	93.33 ± 6.46	99.9 ± 5.92
	100	3.62	109.92	3.41	99.37	68.46 ± 9.43	94.11 ± 10.53
	800	4.17	106.86	3.78	104.32	113.91 ± 1.46	101.72 ± 1.63
Rg1	12.5	5.82	106.52	2.34	100.25	113.38 ± 5.41	103.5 ± 1.52
	100	10.7	106.53	3.21	97.1	85.79 ± 4.83	92.1 ± 2.45
	800	4.62	107.28	2.91	102.65	112.05 ± 6.55	104.48 ± 6.34
Rg2	12.5	7.47	98.55	4.11	107.28	108.67 ± 6.31	115.33 ± 7.84
	100	3.66	101.27	3.19	104.54	103.21 ± 4.13	90.4 ± 3.75
	800	3.58	101.26	3.03	90.01	114.88 ± 7.31	107.34 ± 4.87
Rg5	12.5	11.78	87.6	5.33	100.19	104.78 ± 2.41	106.34 ± 1.53
	100	4.29	94.55	2.15	102.25	92.82 ± 6.31	85.24 ± 2.85
	800	4.67	99.11	3.65	98.5	110.6 ± 2.49	120.04 ± 1.89
Rk1	12.5	4.87	99.67	4.73	102.74	100.98 ± 8.48	101.44 ± 5.06
	100	7.19	98.78	1.5	101.41	85.83 ± 9.62	92.03 ± 7.84
	800	11.14	91.42	3.59	94.75	101.96 ± 7.43	94.93 ± 5.83
Ro	12.5	5	105.06	4.67	100.28	101.49 ± 4.63	99.17 ± 1.24
	100	8.72	103.56	6.28	96.68	85.33 ± 3.74	90.05 ± 4.18
	800	4.48	110.36	7.58	103.04	100.56 ± 5.72	105.19 ± 9.45

**TABLE 4:** Stability of 15 ginsenosides.

Analytes	Spiked QC (ng/mL)	25 °C, 4 h	4 °C, 7 days	−80 °C, 30 d	Freeze-thaw
Rb1	25	−8.93	7.58	0.38	−4.73
	200	−3.11	−13.64	−12.11	−6.81
	1,600	−11.76	−2.14	−10.38	−4.82
Rb2	12.5	−0.68	−4.73	10.05	3
	100	0.07	11.15	6.15	1.53
	800	−10.77	−7.37	4.08	−5.55
Rb3	12.5	4.5	1.58	7.88	7.2
	100	−2.02	10.88	3.1	0.78
	800	−13.02	−10.59	−1.54	−9.18
Rc	12.5	5.48	3.53	11.18	4.35
	100	−2.28	10.01	3.87	0.22
	800	−7.57	−1.28	−1.93	−10.57
Rd	25	−4.58	−12.9	12.6	14.7
	200	−10.31	−7.1	6.16	13.34
	1,600	−11.65	−4.46	4.47	2.58
Ra1	25	6.21	3.81	−13.83	10.78
	200	5.83	−14.02	8.04	−9.62
	1,600	−4.82	6.42	10.74	5.82
Rg3	12.5	11.63	8.55	12.38	8.78
	100	5.09	4.61	5.27	12.35
	800	−11.17	−9.48	6.21	−3.22
CK	12.5	9.87	7.52	5.12	8.64
	100	−7.69	9.62	3.71	−2.81
	800	−8.46	8.45	1.73	−9.42
Re	12.5	0.53	−0.15	12.45	9
	100	5.5	−2.23	13.85	11.37
	800	−5.14	−5.52	10.28	0.97
Rf	12.5	−6	−7.8	−1.5	13.05
	100	−1.74	−6.23	3.99	8.89
	800	−1.62	−9.87	3.72	6.04
Rg1	12.5	−3.75	−2.25	6.3	11.25
	100	−3.25	−8.69	13.18	11.02
	800	−4.21	−4.28	10.1	10.42
Rg2	12.5	9.08	9.45	14.03	8.63
	100	2.99	10.25	9.93	13.2
	800	−14.89	−6.76	−4.44	−1.88
Rg5	12.5	8.63	7.43	2.48	13.67
	100	7.45	6.32	4.61	6.56
	800	4.67	1.83	3.85	−0.82
Rk1	12.5	−0.21	0.05	5.13	10.51
	100	2.79	2.85	6.75	4.38
	800	1.45	7.62	1.54	2.64
Ro	12.5	1.35	−2.85	11.33	6.38
	100	−4.03	7.76	13.07	12.67
	800	2.89	−0.06	14.65	2.73

### 3.3 Comparative pharmacokinetic profiles of total ginsenosides in young and aging mice

**FIGURE 3:**
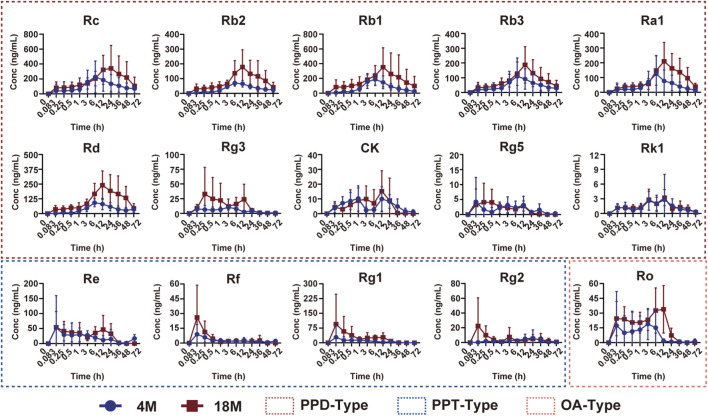
Mean plasma concentration time profiles of 15 major ginsenosides after oral administration of TGs in young versus aging mice. Data are presented as mean ± SD (n = 6 per group).

To characterize the pharmacokinetics of the absorbed ginsenosides into the plasma of C57BL/6 J mice, mice received a single oral dose of TGs (200 mg/kg). The chemical composition of the TGs was quantified, confirming the presence of major protopanaxadiol (PPD), protopanaxatriol (PPT), and oleanolic acid (OA) type ginsenosides, while the metabolite Compound K (CK) was absent in the extract (Supplementary Table S3). Subsequently, 15 relatively abundant ginsenosides and metabolites in plasma were selected to describe the multi-component pharmacokinetic profiles (Figure 3; Table 5). The 15 ginsenosides can be divided into two types: ginsenoside Rb1, Rb2, Rb3, Rc, Rd, CK, Ra1, Rg5, Rk1 and Rg3 are protopanaxadiol type (PPD-type), ginsenoside Re, Rg1, Rg2, and Rf are the protopanaxatriol type (PPT-type). Additionally, Ro belongs to the oleanolic acid type (OA-type). Analysis of the plasma concentration-time curve and pharmacokinetic parameters revealed distinct pharmacokinetic profiles of diol, triol, and oleanolic acid-type ginsenosides exhibit variations following oral administration. Generally, PPT-type ginsenosides exhibited rapid absorption with a short time to reach maximum concentration (T_max_ averaging 4.73 h). In contrast, PPD-type ginsenosides showed significantly slower absorption, with an average T_max_ of approximately 12 h (Figure 3). Additionally, ginsenoside Ro and ginsenoside Rg3 exhibited double peak phenomenon. Notably, while structural types influenced absorption speed, no significant differences in T_max_ were observed for any individual ginsenoside between young and aging mice (Table 5). Despite no significant differences in T_max_, the systemic exposure (as reflected by AUC and C_max_) to ginsenosides Rd, Rb2, Ra1, Rb3, Rc, Rf, and Rb1 was significantly higher in aging mice than in young mice (Table 5). Conversely, only a few components (Rk1, Rg2, and the metabolite CK) showed higher exposure in the young mice. Furthermore, aging mice generally exhibited decreased clearance (CL) and prolonged elimination half-lives (T_1/2_, ranging from 6.1 to 90.05 h) for most ginsenosides. Overall, Aging mice showed increased systemic exposure, decreased clearance, and prolonged half-lives.

**TABLE 5:** Comparative pharmacokinetic parameters of 15 ginsenosides in young and aging mice after oral administration.

Analytes	T_1/2_ (h)		T_max_ (h)		C_max_ (ng/mL)		AUC_0-t_ (h*ng/mL)		AUC_0-_ ∞ (h*ng/mL)		CL (mL/h)		Vc (mL)	
​	18 M	4 M	18 M	4 M	18 M	4 M	18 M	4 M	18 M	4 M	18 M	4 M	18 M	4 M
Rb1	25.65 ± 13.73	28.84 ± 22.14	10.80 ± 8.64	6.50 ± 2.95	379.49 ± 263.58	209.89 ± 86.53	14,300.74 ± 14,743.55*	5,450.93 ± 2,739.62	19,199.61 ± 20,709.49**	7,034.67 ± 4,485.14	26.18 ± 23.28	45.68 ± 48.86	693.75 ± 477.99	1,285.99 ± 716.89
Rb2	29.37 ± 7.38	55.04 ± 36.34	15.60 ± 11.70	8.00 ± 3.10	198.48 ± 110.77	75.60 ± 17.02	7,470.90 ± 5,180.25***	2,785.80 ± 826.41	7,761.57 ± 6,724.15	5,015.93 ± 2,943.35	29.85 ± 24.36	30.44 ± 13.21	1,142.39 ± 816.93	1989.07 ± 681.24
Rb3	31.35 ± 16.69	37.81 ± 23.8	12.60 ± 7.47	10.00 ± 3.10	217.09 ± 113.57	121.04 ± 120.03	7,030.01 ± 4,575.76	3,883.44 ± 3,676.75	9,539.31 ± 6,949.23*	5,738.76 ± 5,589.29	15.11 ± 13.100	31.19 ± 34.43	508.65 ± 434.94	1,124.09 ± 603.02
Rc	35.15 ± 15.08	47.14 ± 18.47	17.40 ± 12.80	8.00 ± 3.10	414.42 ± 279.30	240.79 ± 207.12	16,877.51 ± 13,688.00	8,344.34 ± 7,438.23	20,732.11 ± 23,449.63	13,162.15 ± 11,190.53	16.26 ± 17.74	13.03 ± 6.77	707.08 ± 635.31	845.34 ± 461.15
Rd	22.63 ± 10.36	40.02 ± 14.38	16.80 ± 10.73	11.00 ± 7.01	265.81 ± 142.11	105.25 ± 39.99	10,405.94 ± 6,702.66***	3,315.55 ± 1874.16	10,151.28 ± 8,141.80*	5,184.08 ± 1,460.30	7.97 ± 4.88	11.00 ± 2.79	240.60 ± 156.59	634.45 ± 279.10
Ra1	23.11 ± 7.98	30.12 ± 24.95	19.20 ± 10.73	7.00 ± 2.45	243.67 ± 128.32	126.19 ± 124.22	8,524.75 ± 4,664.48***	3,312.06 ± 2,838.40	9,548.76 ± 6,128.73***	4,044.29 ± 3,215.65	2.96 ± 3.22	5.62 ± 4.00	88.27 ± 78.28	206.95 ± 158.62
Rg3	23.97 ± 22.21	35.40 ± 5.06	7.85 ± 15.79	10.58 ± 8.31	31.80 ± 33.83	11.53 ± 9.14	198.46 ± 253.38	190.13 ± 177.43	103.41 ± 14.68	148.48 ± 59.52	144.13 ± 20.46	108.05 ± 43.31	4,655.74 ± 3,911.43	5,359.43 ± 1,423.45
CK	22.43 ± 13.93	16.89 ± 8.36	12.60 ± 7.47	6.46 ± 9.72	23.82 ± 13.08	16.05 ± 6.17	271.66 ± 144.72	324.22 ± 199.23	323.90 ± 166.26	369.87 ± 226.07	0.30 ± 0.16	0.48 ± 0.66	11.26 ± 12.23	7.49 ± 5.21
Re	9.17 ± 5.73	89.18 ± 97.52	6.03 ± 5.96	30.51 ± 33.20	76.90 ± 50.76	77.93 ± 96.29	1,105.46 ± 899.89	818.99 ± 308.56	1,335.42 ± 907.81*	2,119.18 ± 848.00	153.30 ± 201.25	42.69 ± 17.08	1800.83 ± 2052.71	4,290.60 ± 3,807.98
Rf	12.14 ± 14.39	68.16 ± 64.59	0.67 ± 1.30	15.07 ± 28.29	21.26 ± 33.14	11.09 ± 8.81	21.56 ± 11.74	112.66 ± 87.59	33.70 ± 24.44****	342.79 ± 287.47	1775.44 ± 2,694.67	294.35 ± 483.30	8,487.89 ± 5,987.13	5,292.29 ± 1,654.29
Rg1	6.1 ± 3.47	15.99 ± 6.18	4.23 ± 4.99	5.69 ± 9.25	116.57 ± 139.82	37.53 ± 56.54	585.31 ± 304.28****	194.00 ± 152.78	708.43 ± 210.19****	175.73 ± 79.16	89.09 ± 26.67	397.32 ± 182.59	881.44 ± 762.14	9,288.55 ± 5,639.36
Rg2	23.97 ± 22.21	35.40 ± 5.06	7.85 ± 15.79	10.58 ± 8.31	31.80 ± 33.83	11.53 ± 9.14	198.46 ± 253.38	190.13 ± 177.43	103.41 ± 14.68	148.48 ± 59.52	310.65 ± 44.11	232.89 ± 93.35	10,035.11 ± 8,430.81	11,551.88 ± 3,068.15
Rg5	8.13 ± 1.96	26.37 ± 20.91	3.35 ± 4.95	9.51 ± 8.55	6.51 ± 5.32	7.45 ± 7.02	47.57 ± 20.64	71.69 ± 44.16	65.43 ± 16.90****	167.58 ± 58.91	96.72 ± 29.19	38.16 ± 13.41	1,189.01 ± 662.19	1,249.48 ± 640.55
Rk1	28.63 ± 16.65	90.05 ± 71.77	6.60 ± 4.93	8.67 ± 8.38	3.61 ± 1.78	4.62 ± 4.35	88.75 ± 51.82	85.30 ± 48.97	117.48 ± 54.29	138.63 ± 49.50	71.21 ± 21.12	60.11 ± 24.57	3,004.00 ± 2,187.84	6,374.53 ± 3,087.77
Ro	15.12 ± 16.07	29.42 ± 32.99	4.87 ± 4.94	3.01 ± 1.87	46.67 ± 24.21	26.33 ± 30.4	620.09 ± 358.27****	190.67 ± 193.54	897.23 ± 346.75****	266.5 ± 225.26	41.13 ± 15.60	188.45 ± 105.15	714.98 ± 523.95	7,169.11 ± 7,032.83

Data are presented as mean ± SD (n = 6 per group). Significant differences between young and aging mice are indicated by asterisks (*P < 0.05, **P < 0.01, ***P < 0.001, ****P < 0.0001).

### 3.4 Tissue distribution profiles of ginsenosides in young and aging mice

**FIGURE 4:**
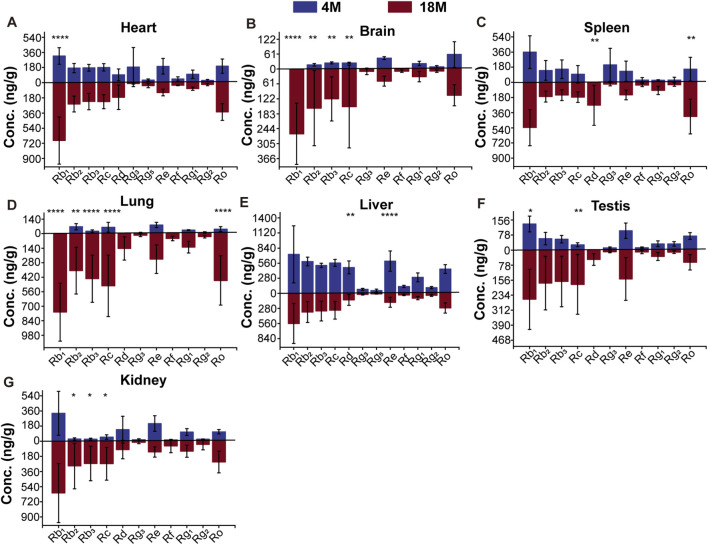
Tissue distribution of 12 ginsenosides in young and aging mice after oral administration of TGs. Data expressed as means ± SD (n = 6). Note: *P ≤ 0.05, **P ≤ 0.01, ***P ≤ 0.001, ****P ≤ 0.0001. **(A-G)** was respectively present different tissue, including heart, brain, spleen, lung, liver, testis and kidney.

To evaluate the early-stage tissue distribution of ginsenosides, tissues were collected at 0.5 h post-administration. Ten major ginsenosides (Rb1, Rb2, Rb3, Rc, Rd, Rg3, Re, Rg1, Rg2, and Rf) were widely detected across all examined tissues, indicating rapid and extensive systemic distribution. The highest concentrations were observed in the liver, followed by highly perfused organs including the heart, kidney, spleen, lung, testis, and brain (Figure 4). Overall, tissue concentrations were lower than those in plasma. Notably, PPD-type ginsenosides exhibited higher tissue accumulation than PPT-type congeners, with ginsenoside Rb1 being the most abundant constituent across all tissues. Additionally, ginsenoside Rg5 showed a highly specific distribution, detected exclusively in the heart and liver. Consistent with the elevated plasma exposure (AUC) observed in the pharmacokinetic analysis, the overall ginsenoside content in the tissues of aging mice was generally higher than that in young mice. Specifically, a characteristic accumulation profile was observed in aging mice, driven primarily by ginsenosides Rb1, Rb2, Rb3, Rc, and Ro. These components exhibited consistently elevated levels across multiple major organs in aging mice, including the brain, lung, kidney, and testis (Figures 4B,D,F,G). Similarly, in the spleen, aging mice showed significantly higher levels of Ro and Rd (Figure 4C, *P* < 0.05). Conversely, a few exceptions were noted where young mice exhibited higher tissue concentrations. For instance, young mice had significantly higher levels of Rd and Re in the liver (Figure 4E, *P* < 0.05), and higher levels of Rg3 in the heart compared to aging mice (Figure 4A).

## 4 Discussions

In this study, we successfully established an 18-month-old natural aging mouse model, characterized by significant declines in physical function, altered body composition (increased fat mass), and impaired cardiac function, consistent with previous reports (Xie and Ehninger, 2023). Utilizing this model, we systematically compared the multi-component pharmacokinetic (PK) and tissue distribution profiles of total ginsenosides between young and aging mice, revealing profound age-related difference after oral administration.

Regardless of age, the absorption profiles of ginsenosides were strongly dictated by their chemical structures. Representative PPT-type ginsenosides (e.g., Re, Rg1) exhibited rapid absorption with short T_max_, whereas PPD-type ginsenosides (e.g., Rb1, Rd) demonstrated significantly delayed absorption. This disparity is primarily attributed to the presence of a C-6 hydroxyl group in the PPT aglycone, which increases molecular polarity and aqueous solubility, thereby facilitating faster gastrointestinal absorption and shorter half-lives compared to the more lipophilic PPD-types (Pan et al., 2018). Furthermore, the double-peak phenomenon observed for specific components like Rg3 and Ro likely results from enterohepatic recirculation or region-specific absorption variability in the gut.

The most striking finding of our study is the significantly elevated systemic exposure (increased AUC and C_max_) and delayed elimination (decreased CL and prolonged T_1/2_) of the majority of ginsenosides in aging mice. Taking the most abundant component, Rb1, as a representative example, its clearance was markedly reduced in the aging mice. Firstly, aging is accompanied by a 20%–50% reduction in hepatic blood flow and a significant decline in the expression and activity of hepatic drug-metabolizing enzymes, particularly the cytochrome P450 (CYP450) system (Shi and Klotz, 2011; Bae et al., 2014). Since the liver is the primary site for ginsenoside metabolism, this age-related hepatic impairment directly leads to reduced first-pass effect and slower systemic clearance, thereby accumulating ginsenosides in the circulation of elderly subjects. Thus, the increased systemic exposure of ginsenosides (Rc, Ra1, Rb1, Rb2, Rb3, Rd, Rg3, Rg1, Ro) in aging mice may be related to first-pass metabolism, which could highlight potential candidates for anti-aging interventions. Besides, according to previous reports, rats received Panax *Notoginseng* saponins (PNS) orally at 40 mg/kg or intravenously at 10 mg/kg (Jeon et al., 2020). In rats, ginsenoside Rb1 reached T_max_ within 4 h. This result differs from findings in mice. The discrepancy may be caused by species-specific differences in absorption.

In addition to hepatic clearance, age-related changes in body composition significantly affect drug distribution. Our phenotypic data showed that aging mice possessed increased body fat and decreased lean mass/total body water. This physiological shift alters the apparent volume of distribution (Vc), particularly favoring the retention of highly lipophilic PPD-type ginsenosides in adipose tissues, further prolonging their elimination half-lives. Moreover, the absorption of ginsenosides is highly dependent on deglycosylation by gut microbiota (Hasegawa, 2004). The altered exposure of specific metabolites, such as CK, between young and aging mice strongly suggests that age-related dysbiosis of the gastrointestinal microbiome plays a crucial role in modulating the biotransformation and subsequent bioavailability of ginsenosides. We also found that the ginsenosides circulating in the blood system are mainly PPD-type ginsenoside Rc and Rb1. The predominance of Rc and Rb1 in circulation reflects their higher abundance in the administered TGs extract compared to other ginsenosides (Table 2). These results indicated that PPD-type ginsenosides might be the main pharmacological active components *in vivo* after oral administration (Fu et al., 2023).

The tissue distribution profile further corroborated the PK findings, showing generally higher ginsenoside accumulation in the organs of aging mice. A critical observation was the differential distribution in the brain. Notably, Rb1 was nearly undetectable in the brain tissue of young mice but accumulated at substantial levels in aging mice. This phenomenon can be mechanistically explained by the age-associated compromise of the blood-brain barrier (BBB) integrity. Aging induces endothelial dysfunction and tight junction degradation, resulting in enhanced BBB permeability that facilitates the entry of large-molecule exogenous compounds into the central nervous system (Lee et al., 2025). Similar age-related increases in vascular permeability likely occur in peripheral tissues (such as the lung and heart), explaining the widespread elevation of ginsenoside tissue retention. Furthermore, aging is commonly associated with reduced plasma albumin levels, which decreases plasma protein binding and increases the free (unbound) fraction of drugs in the blood (Ngcobo, 2025). This elevated free drug availability creates a larger concentration gradient, driving more ginsenosides from the systemic circulation into target tissues, thereby potentially amplifying both their pharmacological efficacy and risk of toxicity in the elderly.

## 5 Conclusion

In summary, this study demonstrated that aging significantly alters the pharmacokinetics and tissue distribution of ginsenosides, characterized by reduced clearance, prolonged half-life, and increased systemic exposure (AUC) in aging mice. Furthermore, age-related compromises in blood-brain barrier integrity were found to facilitate the cerebral accumulation of specific ginsenosides like Rb1. These findings provide a mechanistic foundation for the differential pharmacological effects of ginseng in the elderly and offer critical insights for its precision application in anti-aging and cardioprotection.

## Data Availability

The original contributions presented in the study are included in the article/Supplementary Material, further inquiries can be directed to the corresponding authors.
