# Supplementary material for: Comparative pharmacokinetics of total ginsenosides between young and aging mice
Source: Front Pharmacol. 2026 Jun 30;17:1844832. doi: 10.3389/fphar.2026.1844832 (PMC13364985; doi:10.3389/fphar.2026.1844832)
Supplement: Supplementary file 1 [file Supplementaryfile1.pdf]

Comparative Pharmacokinetics of Total Ginsenosides between Young and Aging Mice

Zuoyang Li[1,2], Nanqi Hou[1,2], Yuying Zhou[1,2], Yan Zhang[2], ZiChen Tian[2], Xinyue Cao[2], Daqing Zhao[2], Hang Su[1,2*], Xiangyan Li[1,2*]

[1]Northeast Asia Research Institute of Traditional Chinese Medicine, Jilin Provincial Key Laboratory for Efficacy Research and Utilization of Characteristic Traditional Chinese Medicine, Changchun University of Chinese Medicine, Changchun, 130117, China.
[2]Department of Endocrinology, The First Affiliated Hospital of Changchun University of Chinese Medicine, Changchun, 130021, China.

*** Correspondence:**
Xiangyan Li, xiangyan_li1981@163.com; Hang Su, suhang0720@live.cn.

Keywords: Ginsenosides, Pharmacokinetics, Tissue distribution, MRM, UPLC-MS/MS

Legend

Figure S1. The levels of oxidative factors and β-galactosidase in various tissues of mice. (A) - (C) are the activity of SOD in heart, liver and brain, respectively. (B) - (D) are MDA levels in heart, liver and brain, respectively. (G) - (I) are β-galactosidase activity in heart, liver and brain, respectively. Data are presented as the mean ± SD of three mice.

Table S1. Optimized multiple reaction monitoring (MRM) parameters. Declustering potential (DP), entrance potential (EP), collision energy (CE), and cell exit potential (CXP) for 7 ginsenosides are listed.

Table S2 Linearity and Limit of Quantification of 22 ginsenosides.

Table S3. The stability of 6 batch TGs and the dosage of each ginsenosides.

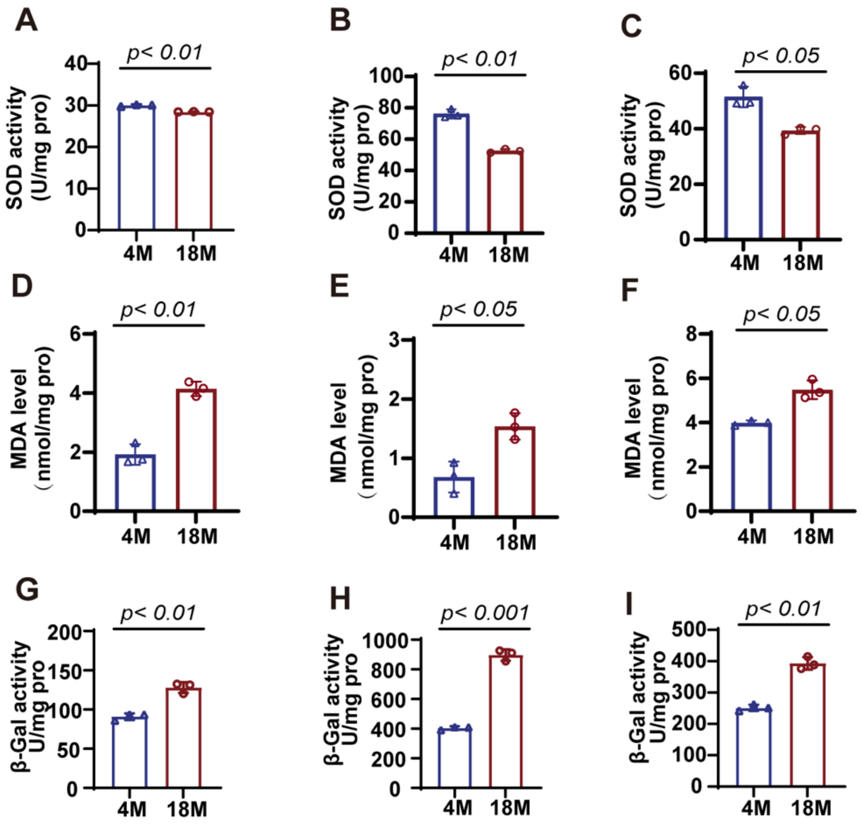

Figure S1 The levels of oxidative factors and β-galactosidase in various tissues of mice. (A) - (C) are the activity of SOD in heart, liver and brain, respectively. (B) - (D) are MDA levels in heart, liver and brain, respectively. (G) - (I) are β-galactosidase activity in heart, liver and brain, respectively. Data are presented as the mean ± SD of three mice.

Table S1 Optimized multiple reaction monitoring (MRM) parameters. Declustering potential (DP), entrance potential (EP), collision energy (CE), and cell exit potential (CXP) for 7 ginsenosides are listed.

Compound	Module	Precursor ion (m/z)	Produc tion (m/z)	DP	CE	EP	CXP
F2	Negative	829.6	621.2	-130	-42	-10	-13
PPT	Negative	475.4	391.3	-150	-40	-10	-13
F1	Negative	637.4	475.3	-150	-30	-10	-13
F5	Negative	769.5	637.6	-160	-35	-10	-13
Rh1	Negative	683.3	637.5	-130	-26	-10	-13
Pf11	Negative	799.6	653.5	-150	-45	-10	-13
20-O-Glc-Rf	Negative	961.5	799.4	-50	-30	-10	-13

Table S2 Linearity and Limit of Quantification of 22 ginsenosides.

analytes	linearity	R^2	Range	LOQ (ng/mL)
F5	y=165.882x + 575.797	0.994	2.63-1250	2.63
F1	y=126.093x + 481.261	0.996	10.55-1250	10.55
pf11	y=1659.986x + -151.030	0.995	0.66-1250	0.66
Ro	y=348.939x + -5478.031	0.997	21.09-1250	21.09
CK	y=4086.751x + -188.290	0.996	0.66-1250	0.66
Rh1	y=3413.746x + 512.423	0.996	0.66-1250	0.66
Re	y=12676.248x + 3451.369	0.998	0.66-1250	0.66
Rd	y=286.592x + 411.343	0.998	1.32-2500	1.32
Rf	y=330.054x + -10.335	0.998	1.32-1250	1.32
Rg1	y=6020.525x + 917.894	0.999	0.66-1250	0.66
F2	y=1106.476x + -127.330	0.998	0.66-1250	0.66
Rg2	y=24594.274x + 4225.926	0.998	0.66-1250	0.66
Rg3	y=17650.666x + 3237.487	0.997	0.66-1250	0.66
Rg5	y=16211.917x + 925.998	0.997	0.66-1250	0.66
Rk1	y=10142.072x + -1343.019	0.997	0.66-1250	0.66
Rb2	y=3607.360x + 472.320	0.998	0.66-1250	0.66
Rb3	y=4236.355x + 240.836	0.999	0.66-1250	0.66
Rc	y=3921.413x + 1922.720	0.992	0.66-1250	0.66
Ra1	y=126.381x + -31.663	0.996	5.27-2500	5.27
PPT	y=110.704x + 483.663	0.997	1.32-1250	1.32
Rb1	y=481.461x + -107.000	0.997	1.32-1250	1.32
20-O-Glc-Rf	y = 143.571x + -3.559	0.999	1.32-1250	1.32

Table S3 The stability of 6 batch TGs and the dosage of each ginsenosides.

analytes	S1	S2	S3	S4	S5	S6	Mean (mg/g)	SD	Dose (mg/g)
Rb1	82.582	95.658	81.388	98.351	110.130	83.876	91.997	11.399	18.399
Rb2	47.249	82.492	74.372	76.713	64.311	55.746	66.814	13.509	13.362
Rd	44.183	68.831	44.502	64.008	76.992	75.739	62.376	14.744	12.475
Rc	50.839	65.331	54.847	63.776	67.595	61.131	60.586	6.481	12.117
Re	39.050	32.261	27.778	26.936	33.682	33.948	32.276	4.458	6.455
Rg3	33.188	53.701	35.122	19.463	19.702	16.562	29.623	14.106	5.924
Ra1	15.156	24.898	19.173	28.829	33.72	26.341	24.686	6.674	4.937
Rg1	22.111	20.386	16.642	17.075	22.103	19.296	19.602	2.383	3.920
Rk1	14.742	24.618	17.107	11.629	14.325	8.491	15.152	5.496	3.030
Rg5	15.593	26.871	19.439	8.567	9.696	7.935	14.684	7.480	2.936
Rf	11.411	16.142	13.22	14.053	14.969	12.162	13.660	1.762	2.732
Ro	11.506	15.574	13.359	12.179	12.375	10.219	12.535	1.816	2.507
Rb3	7.999	11.110	8.722	11.815	11.577	9.836	10.176	1.580	2.035
F5	9.854	7.924	9.072	10.596	11.607	9.162	9.702	1.288	1.940
Rg2	1.483	2.032	2.132	1.773	1.519	1.306	1.707	0.327	0.341
F1	0.165	0.283	0.237	0.136	0.266	0.097	0.197	0.075	0.039
Rh1	0.152	0.445	0.179	0.135	0.148	0.102	0.193	0.125	0.038
F2	0	0	0	0	0	0	0	0	0
20-O-Glu-Rf	0	0	0	0	0	0	0	0	0
Pf11	0	0	0	0	0	0	0	0	0
CK	0	0	0	0	0	0	0	0	0
PPT	0	0	0	0	0	0	0	0	0
